# Break the Interacting Bridge between Eu^3+^ Ions in the 3D Network Structure of CdMoO_4_: Eu^3+^ Bright Red Emission Phosphor

**DOI:** 10.1038/s41598-018-24374-3

**Published:** 2018-04-12

**Authors:** Weiguang Ran, Hyeon Mi Noh, Sung Heum Park, Byung Kee Moon, Jung Hyun Jeong, Jung Hwan Kim, Jinsheng Shi

**Affiliations:** 1 Department of Physics, Pukyong National University, Busan, 608–737 South Korea; 2 Department of Physics, Dongeui University, Busan, 614-714 South Korea; 3 Department of Chemistry and Pharmaceutical Science, Qingdao Agricultural University, Qingdao, 266109 People’s Republic of China

## Abstract

Eu^3+^ doped CdMoO_4_ super red emission phosphors with charge compensation were prepared by the traditional high temperature solid-state reaction method in air atmosphere. The interrelationships between photoluminescence properties and crystalline environments were investigated in detail. The 3D network structure which composed by CdO_8_ and MoO_4_ polyhedra can collect and efficiently transmit energy to Eu^3+^ luminescent centers. The relative distance between Eu^3+^ ions decreased and energy interaction increased sharply with the appearance of interstitial occupation of O^2−^ ions (${O^{\prime\prime} }_{i}$). Therefore, fluorescence quenching occurs at the low concentration of Eu^3+^ ions in the 3D network structure. Fortunately, the charge compensator will reduce the concentration of ${O^{\prime\prime} }_{i}$ which can break the energetic interaction between Eu^3+^ ions. The mechanism of different charge compensators has been studied in detail. The strong excitation band situated at ultraviolet and near-ultraviolet region makes it a potential red phosphor candidate for n-UV based LED.

## Introduction

White light-emitting diodes (WLEDs), as the promising next generation of illumination sources to replace conventional incandescent and fluorescent lamps, have attracted much attention due to their high efficiency, long lifetime and environment-friendly characteristics^1,2^. Phosphor-converted light-emitting diode (pc-LED) is the mainstream technology to produce solid-state illumination devices^3,4^. Nowadays, the commercial and excellent approach is the combination of cerium-doped yttrium aluminum garnet (YAG:Ce^3+^) phosphor and blue InGaN LED chip which was first proposed in1997^5^. However, there are some disadvantages owing to the innate deficiency of this design such as poor color rendering ability, low chromatic stability under different driving currents, low efficiency and low color rendering index^6,7^. Therefore, white LEDs which fabricated by near-UV LED chips and tri-color phosphors might conquer the aforementioned pitfalls^2,8^. Meanwhile, with the development of LED chip technology, the emission bands of LED chips shifted from blue light to near-UV range which can offer higher energy to pump the phosphor^9^. For the above reasons, more and more attentions have been paid on phosphors with high absorption in the near-UV spectral region^10–12^.

Cadmium molybdate CdMoO_4_ with scheelite-type structure have attracted considerable interest due to its potential applications in phosphors, scintillators, magnets and catalysts^13,14^. Eu^3+^-doped metal molybdates is a kind of potential red emitting phosphor due to their predominant ^5^D_0_ → ^7^F_2_ transition emission, whereas the other emissions are very weak^15^. It is reported that CdMoO_4_:Eu^3+^ shows not only desirable absorption in the near-UV region, but also excellent thermal and chemical stability^16–18^.

However, to the best of our knowledge, there are only a few reports on CdMoO_4_:Eu^3+^ phosphors, whether on its synthesis process or properties. For example, the optimum doping concentration of Eu^3+^ in Cd_1−x_Eu_x_MoO_4_ phosphor prepared through microwave assisted synthesis is 5%^19^. Wang^16^ has prepared an intense CdMoO_4_:Eu^3+^ red emission phosphor through a facile aqueous solution route at room temperature. Guzik^18^ has prepared scheelite-type Cd_1–3x_Eu_2x_□_x_MoO_4_ phosphors by high temperature solid phase reaction method with CdMoO_4_ and Eu_2_(MoO_4_)_3_ as start materials. And the effect of Cd^2+^ vacancy on the occupied position of doping ions in CdMoO_4_ was investigated for the first time^18,20^.

In this paper, a series of Cd_1−x−y_MoO_4_:xEu^3+^, yM (M = Li^+^, Na^+^, K^+^ ions and Cd^2+^ vacancy) phosphors have been prepared via a high temperature solid-state reaction process and their structural and photoluminescence properties were thoroughly investigated. The geometry optimization and electronic structure calculations were performed using the Cambridge Serial Total Energy Package (CASTEP) code^21^. The relationship between the photoluminescence properties and lattice environments of the doped Eu^3+^ ions with different charge compensators was discussed in detail. As the phosphor was pumped by near UV light, superior color purity and high red emission intensity with chromaticity coordinates (0.6451, 0.3451) was achieved. The charge compensation model in this 3D network structure was expected to be used to design efficient luminescent materials.

## Results

### Crystal Structures, Phase Identification and Purity

**Figure 1:**
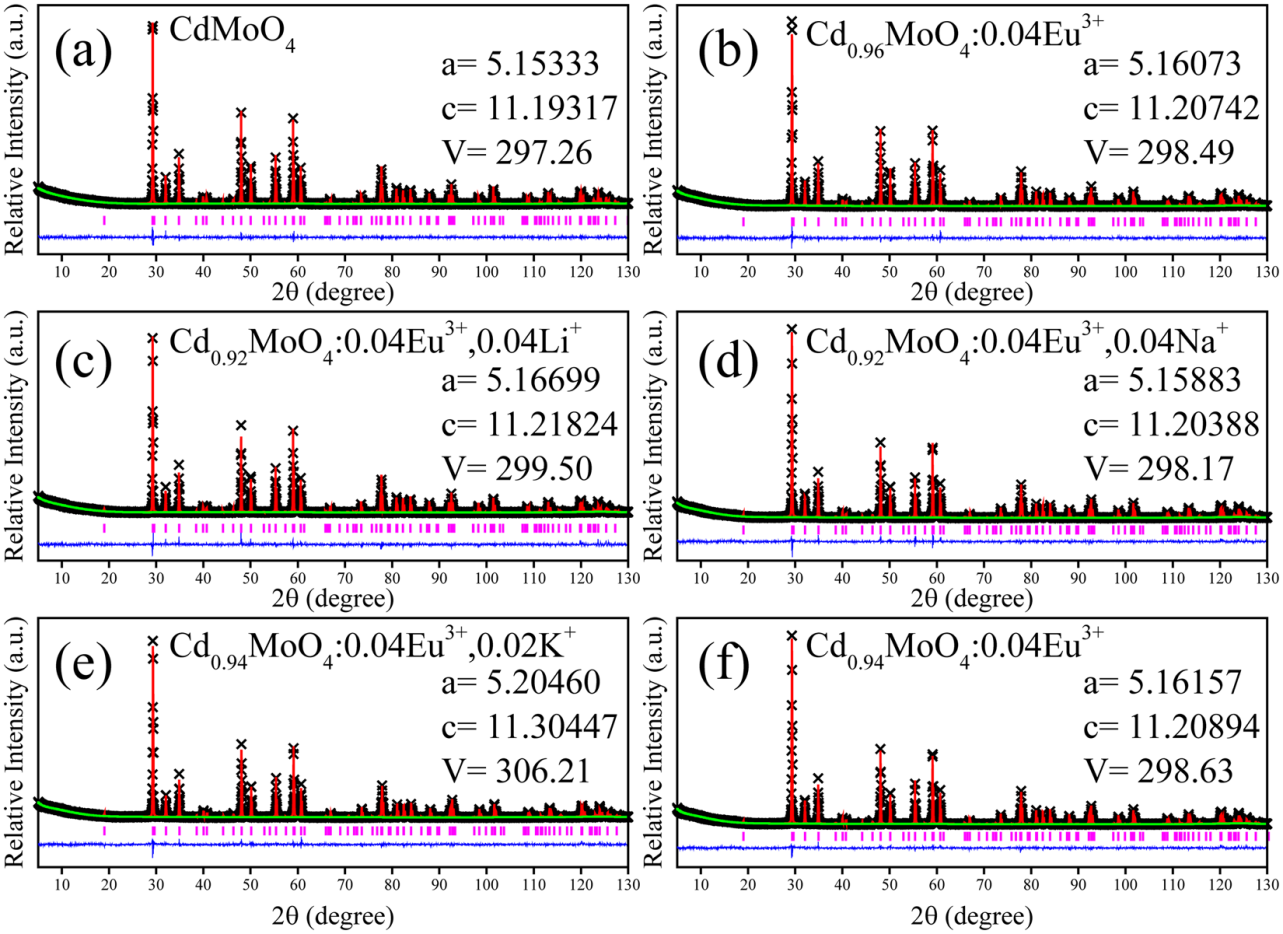
Final Rietveld refinement profiles of CdMoO_4_, Cd_0.96_MoO_4_:0.04Eu^3+^, Cd_0.96−y_ MoO_4_:0.04Eu^3+^, yM (M = Li^+^, Na^+^, K^+^ ions and Cd^2+^ vacancy) phosphors.

The structure refinement of the CdMoO_4_, Cd_0.96_MoO_4_:0.04Eu^3+^ and Cd_0.96−y_MoO_4_:0.04Eu^3+^, yM (M = Li^+^, Na^+^, K^+^ and Cd^2+^ vacancy) samples was performed using the GSAS II software package^22^ and the initial parameters of refinement for CdMoO_4_ sample were referred from the single crystal data of CdMoO_4_ (ISCD-84455). All atomic positions, fraction factors and temperature factors were refined convergence and well satisfied the reflection condition. The final refinement patterns are illustrated in Fig. 1. It can be seen from Fig. 1 that the incorporation of Eu^3+^ and charge compensators affect the unit cell parameters. Cell volumes were increased with the introduction of Eu^3+^ ions. This lattice distortion occurred due to the differences of ionic radius and valence states. It appears from Fig. 1 that the unit cell parameters were effectively regulated with different charge compensators. And the introduction of Na^+^ ions can minimize the lattice distortion.

**Figure 2:**
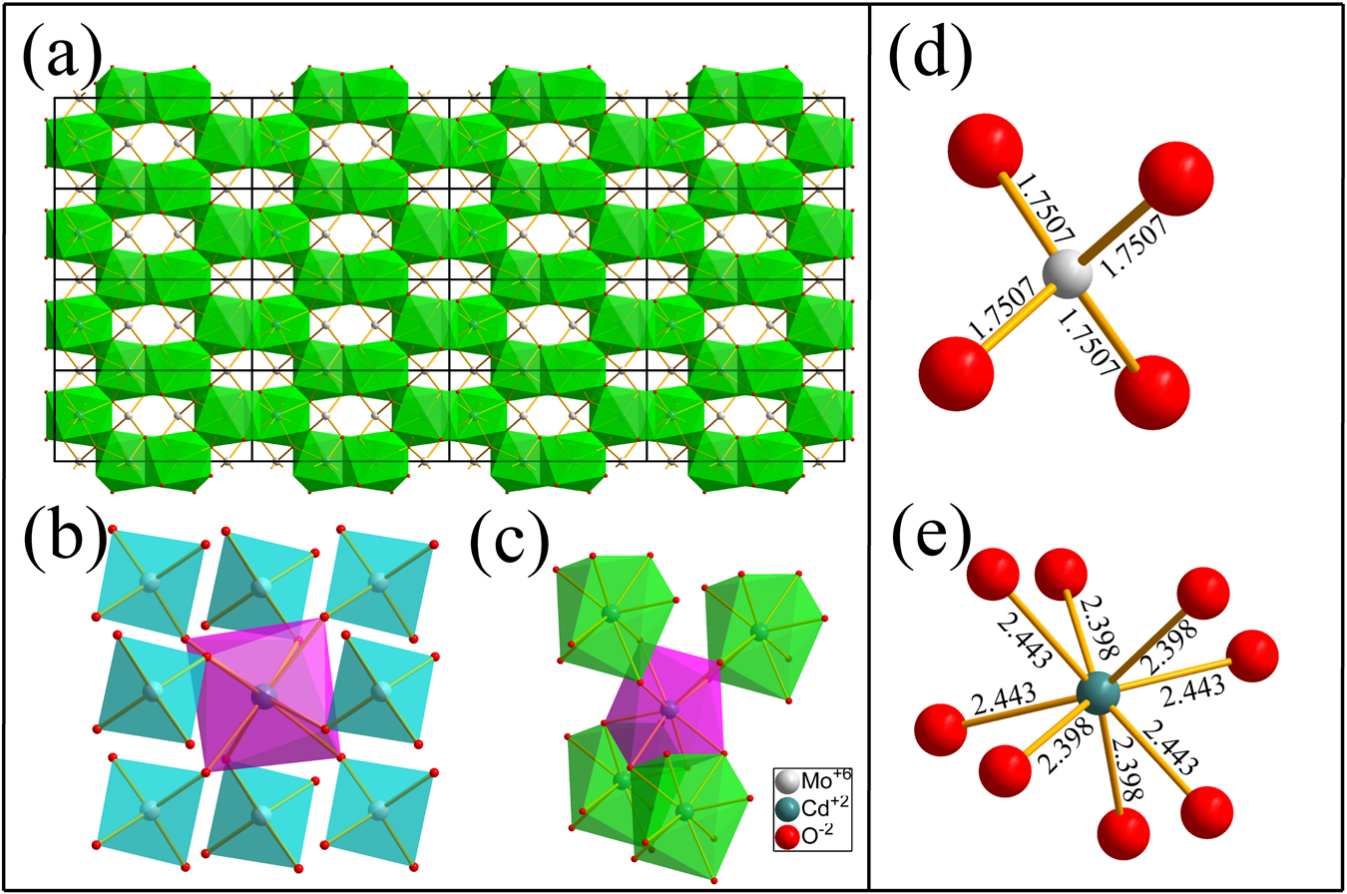
The structure of CdMoO_4_ viewed along different directions. The green polyhedron represents Cd^2+^ center and the blue polyhedron represents the Mo^6+^ center.

The structure model of CdMoO_4_ with a 4 × 4 × 4 supercell along *a*-direction (same with *b*-direction) was shown in Fig. 2(a). Green cross chains were interconnected to form a 3D network structure. From Fig. 2(b), each CdO_8_ dodecahedron connects with eight MoO_4_ tetrahedra units by common vertex connection. It can be seen from Fig. 2(c) that one CdO_8_ is surrounded by four CdO_8_ dodecahedrons and become a tetrahedral structure by sharing common edge between each other. The three-dimensional framework in CdMoO_4_ was composed of CdO_8_ dodecahedron and MoO_4_ tetrahedron. All of CdO_8_ and MoO_4_ polyhedra are connected to form a 3D network structure while MoO_4_ fill in the interspace. From Fig. 2d and e, the coordination environment of Mo^6+^ and Cd^2+^ ions is intuitively displayed. Four coordinated Mo sites have the same Mo-O distance even eight Cd-O bonds lengths are approximate.

### First-principles Calculation and Band Structures

**Figure 3:**
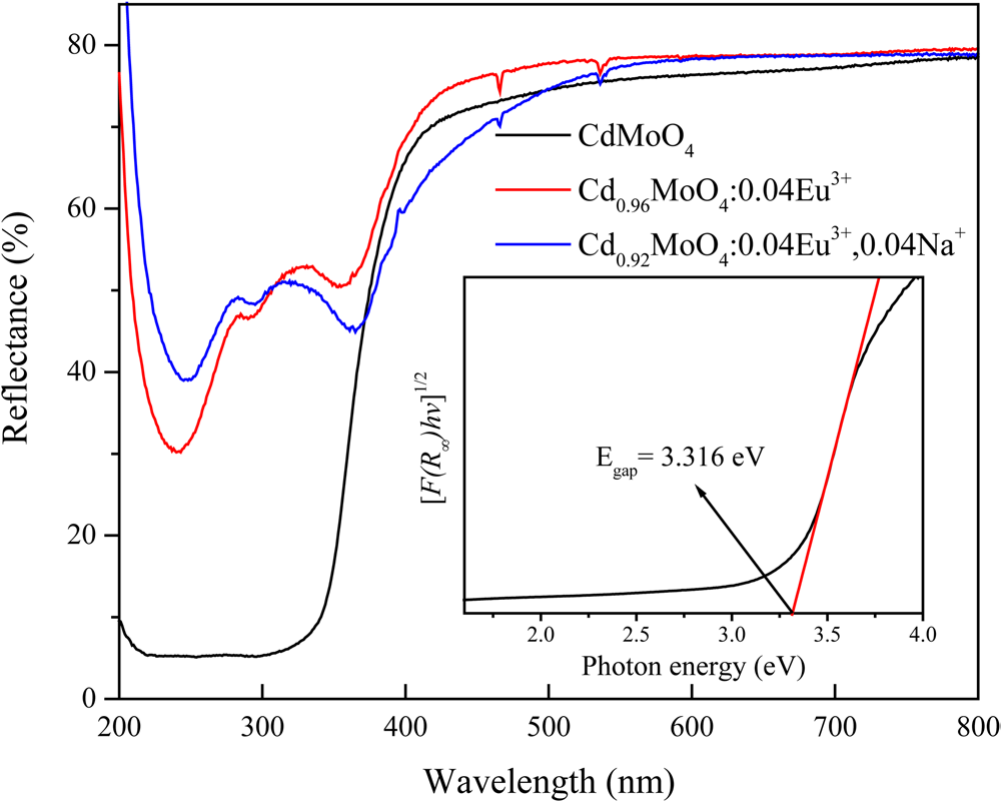
UV-vis diffuse reflection spectra of the as-synthesized CdMoO_4_, Cd_0.96_MoO_4_:0.04Eu^3+^ and Cd_0.92_MoO_4_:0.04Eu^3+^, 0.04Na^+^ phosphors.

The light absorption capability of the as prepared samples of CdMoO_4_, Cd_0.96_MoO_4_:0.04Eu^3+^ and Cd_0.92_MoO_4_:0.04Eu^3+^, 0.04 Na^+^ have been evaluated using the reflectance from BaSO_4_ as a reference. It is shown in the UV-vis diffuse reflectance spectra in Fig. 3 that these samples have strong absorption band in UV and near UV light region. For CdMoO_4_ host, the correlation between the optical band gap ${E}_{gap}$ and absorption coefficient of semiconductor oxides can be determined by the following equation^23,24^:

1 $$F({R}_{\infty })hv\propto {(hv-{E}_{gap})}^{n}$$

2 $$F({R}_{\infty })=\frac{{(1-{R}_{\infty })}^{2}}{2{R}_{\infty }}$$

where $\,v\,\,$is the photon energy, ${R}_{\infty }$ is the reflectivity of the sample, $h$ is the Plank constant, and $n$ is determined by the transition type ($n$ = 1/2, 2, 3/2 or 3 for allowed direct, allowed indirect, forbidden direct and forbidden indirect electronic transitions, respectively).

**Figure 4:**
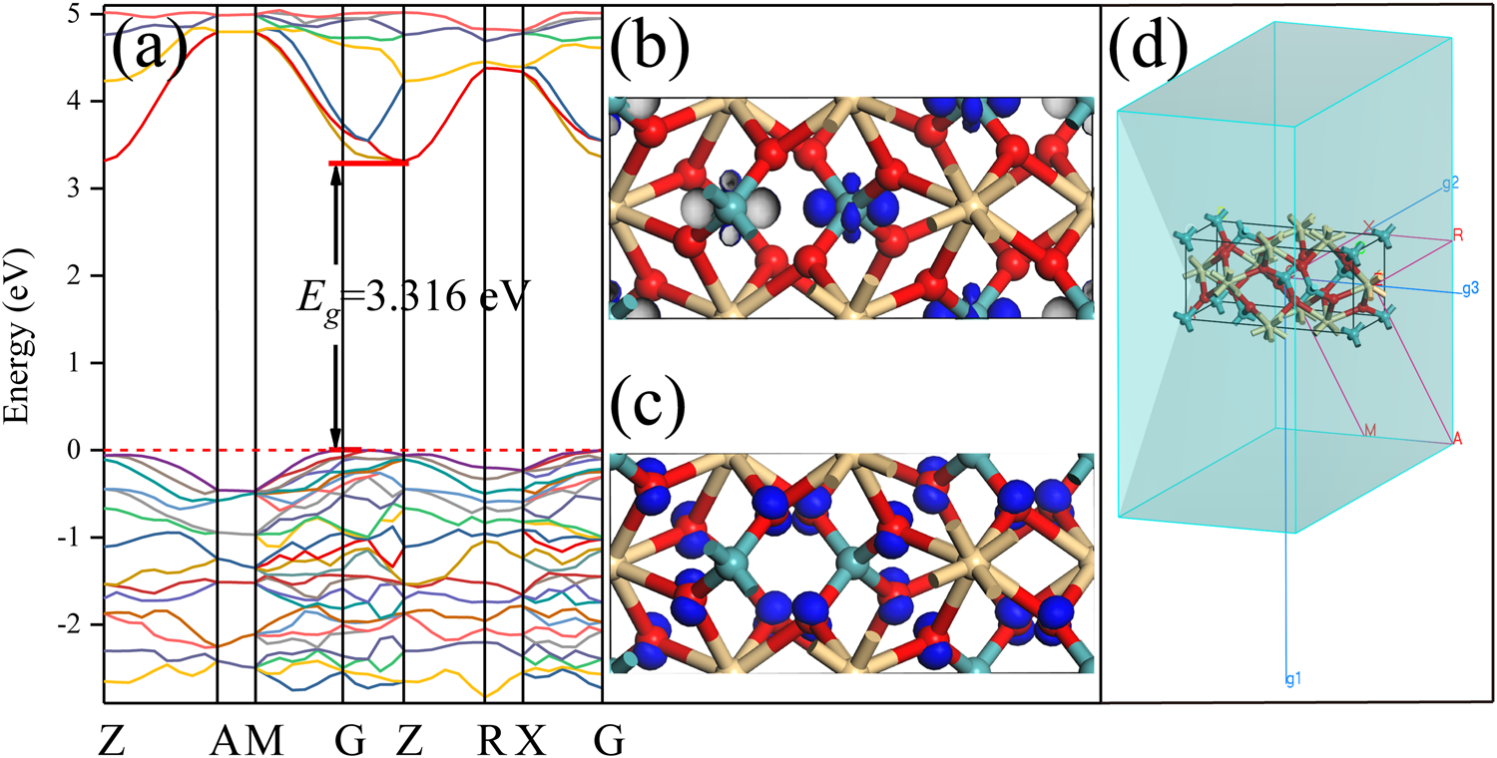
Calculated electronic band structure (**a**); electron orbitals of excited (**b**) and ground (**c**) states of CdMoO_4_ near the Femi energy level (EF); and the Brillouin zone path for band structure calculation (**d**). The Femi energy is the zero of the energy scale.

According to the calculation results of electronic band structure in Fig. 4, the semi-conducting character of CdMoO_4_ has been confirmed to be allowed indirect band gap material, therefore, $n$ is equal to 2.

3 $$hv-{E}_{gap}\propto {[F({R}_{\infty })hv]}^{1/2}$$

As can be seen from the Tauc-plots of CdMoO_4_ sample in the inset of Fig. 3, by extrapolating the linear fitted regions to ${[F({R}_{\infty })hv]}^{1/2}=0$, the absorption boundary is 3.316 eV.

For the samples with Eu^3+^ doped, the weak absorption in the visible light region is consistent with the white color of powders for the naked eye. And compared with the commercial red emitting phosphors, CdMoO_4_:Eu^3+^ phosphors can effectively avoid the disadvantages such as reabsorption in the visible light region.

In order to figure out the origin of the broad band absorption in the UV-vis diffuse reflection spectra of Fig. 3, the calculation of the electronic structure for CdMoO_4_ host was carried out using CASTEP code by density functional theory. The Brillouin zone path for the band structure calculation was shown in Fig. 4(d) and Table 1. The calculated electronic band structure and electron orbitals are given in Fig. 4(a). The lowest energy (2.361 eV) of conduction band (CB) located at Z site, and the valence band (VB) maximum (0.00 eV) is at G site of Brillouin zone, indicating that CdMoO_4_ is an indirect band gap compound and the energy band gap is about 2.361 eV. It is well known that the DFT calculations with CASTEP program generally underestimate the band-gap energies of the semiconductor materials due to the limited dimension of the atomic cluster. Therefore, a scissors operator of 0.955 eV was introduced to widen the gap to consistent with the measured optical band gap value (3.316 eV) of CdMoO_4_ host.

**Table 1:** Brillouin Zone Path.

From				To			
Z	0.000	0.000	0.500	A	0.500	0.500	0.500
A	0.500	0.500	0.500	M	0.500	0.500	0.000
M	0.500	0.500	0.000	G	0.000	0.000	0.000
G	0.000	0.000	0.000	Z	0.000	0.000	0.500
Z	0.000	0.000	0.500	R	0.000	0.500	0.500
R	0.000	0.500	0.500	X	0.000	0.500	0.000
X	0.000	0.500	0.000	G	0.000	0.000	0.000
Z	0.000	0.000	0.500	A	0.500	0.500	0.500

After first-principle calculation, the electron orbitals were shown in Fig. 4b and c. It is well known that the ${\rm{d}}$ orbital splits into ${d}_{xy}$, ${d}_{xz}$, ${d}_{yz}$, ${d}_{{z}^{2}}$ and ${d}_{{x}^{2}-{y}^{2}}$ in the tetrahedral crystal field due to the Jahn-Teller effect. The electron orbitals of the Mo atoms in the tetrahedron crystal field are shown in the Fig. 4(b). It can be seen that the excited electrons enter into the ${d}_{{z}^{2}}$ and ${d}_{{x}^{2}-{y}^{2}}$ orbitals with lowe energy. From Fig. 4(b), the states in the conduction band region are dominated by Mo 4d orbitals. Figure 4(c) shows the position of electrons in the valance band region. The shape of electron distribution is two hemispheres which belong to the typical 2p orbitals of O atoms. The diagram of electron orbitals illustrates that the top of VB is mainly composed by 2p orbital of O atom. Therefore, the absorption bands in Fig. 3 are ascribed to electron transitions from oxygen 2p orbital to an empty molybdenum 4d orbital.

**Figure 5:**
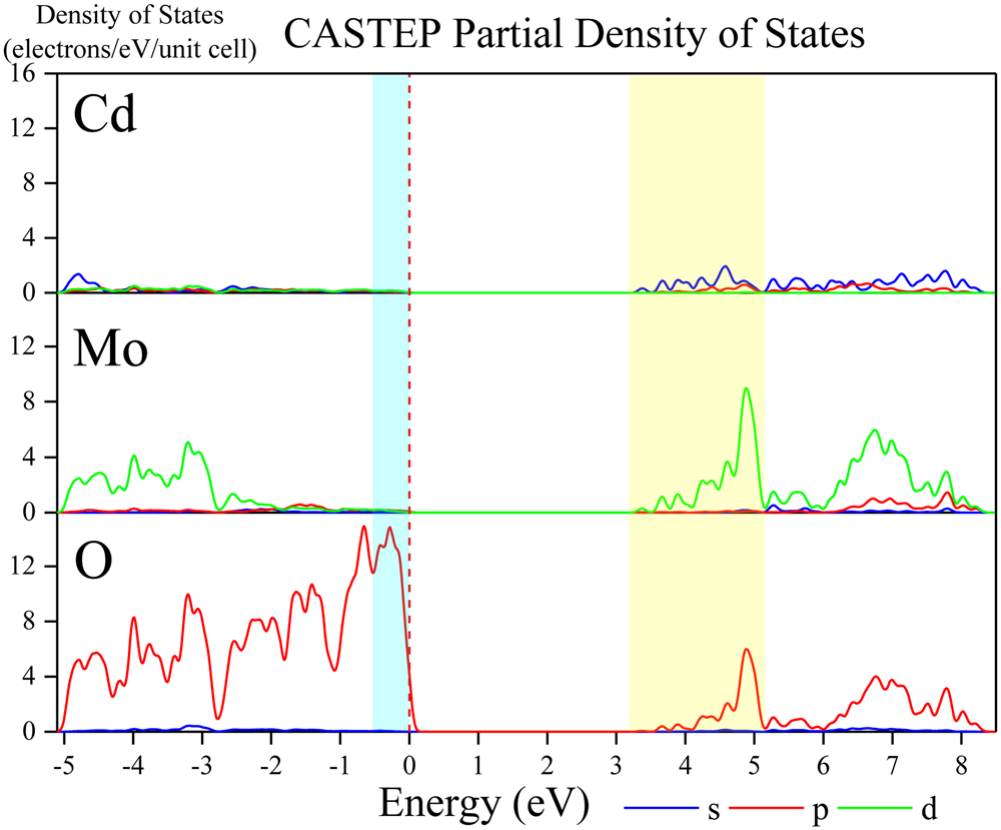
Diagrams of PDOS of Cd/Mo/O for CdMoO_4_ host.

Taking into account the lower display resolution of the electron orbitals diagram, theoretical calculations on the partial density of states (PDOS) for CdMoO_4_ host were carried out and the results are illustrated in Fig. 5. From Fig. 5, the valence bands are dominated by the O 2p states. The integral area of 5s orbitals in the excited states is large enough to be taken into account. It means that the 5s orbitals of Cd atom is an important part of the charge transfer band. The strong hybridization appears between Cd 5s, Mo 4d and O 2p states in the bottom of the conduction bands. The strong absorption bands in Fig. 3 are mainly originated from the charge transfer bands from O 2p to Mo 4d and Cd 5s orbitals.

### Photoluminescence properties

**Figure 6:**
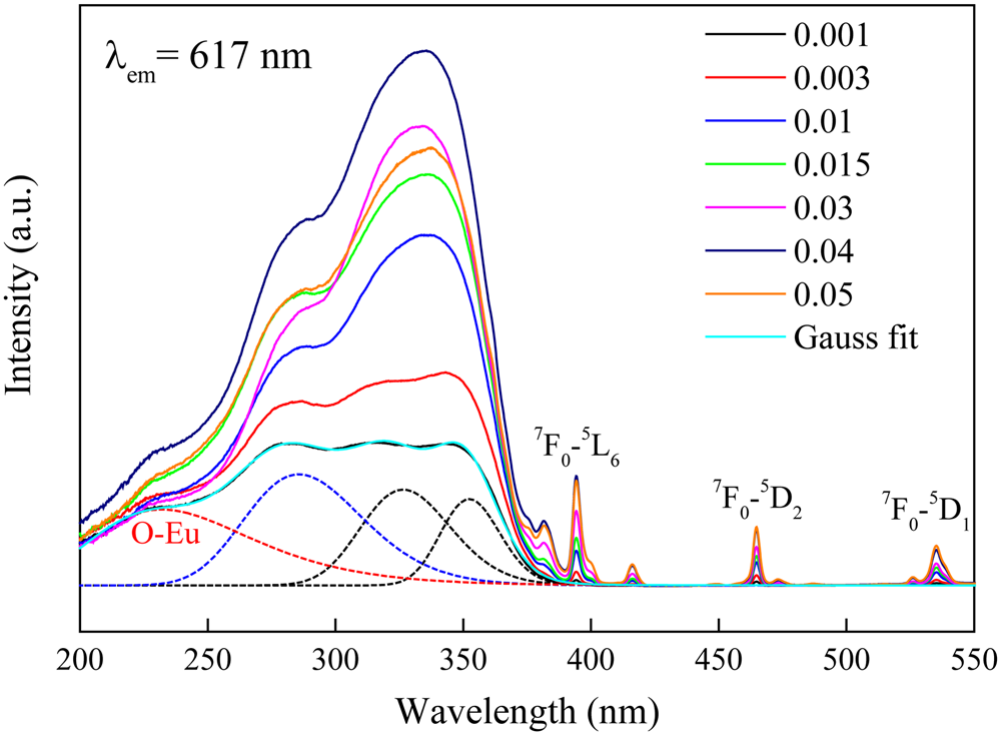
The excitation spectra of Cd_1−x_MoO_4_:xEu^3+^ phosphor monitored at 617 nm emission (^5^D_0_ − ^7^F_2_) at room temperature.

A series of Cd_1−x_MoO_4_:xEu^3+^ samples were synthesized to optimize the doping concentration of Eu^3+^ ions. Figure 6 shows the excitation spectra of Cd_1−x_MoO_4_:xEu^3+^ phosphors monitored at ^5^D_0_ − ^7^F_2_ (617 nm) emission of Eu^3+^ ions. The broad excitation bands ranging from 200 nm to 380 nm are related to the charge transfer transition of O^2−^ → Mo^6+^ and O^2−^ → Eu^3+^. According to the reported literature, the sharp peaks at 394 and 465 nm are due to ^7^F_0_ − ^5^L_6_ and ^7^F_0_ − ^5^D_2_ transitions of Eu^3+^, respectively. When the doping concentration of Eu^3+^ was less than 4%, the relative intensity of excitation bands increases with the increase of Eu^3+^ concentration. A more interesting thing is that the relative intensity of excitation bands, especially at about 328 nm, enhanced sharply with the increase of Eu^3+^ concentration. However, when the doping concentration of Eu^3+^ was beyond 4%, the intensity of excitation bands decreased with increasing Eu^3+^ concentration.

**Figure 7:**
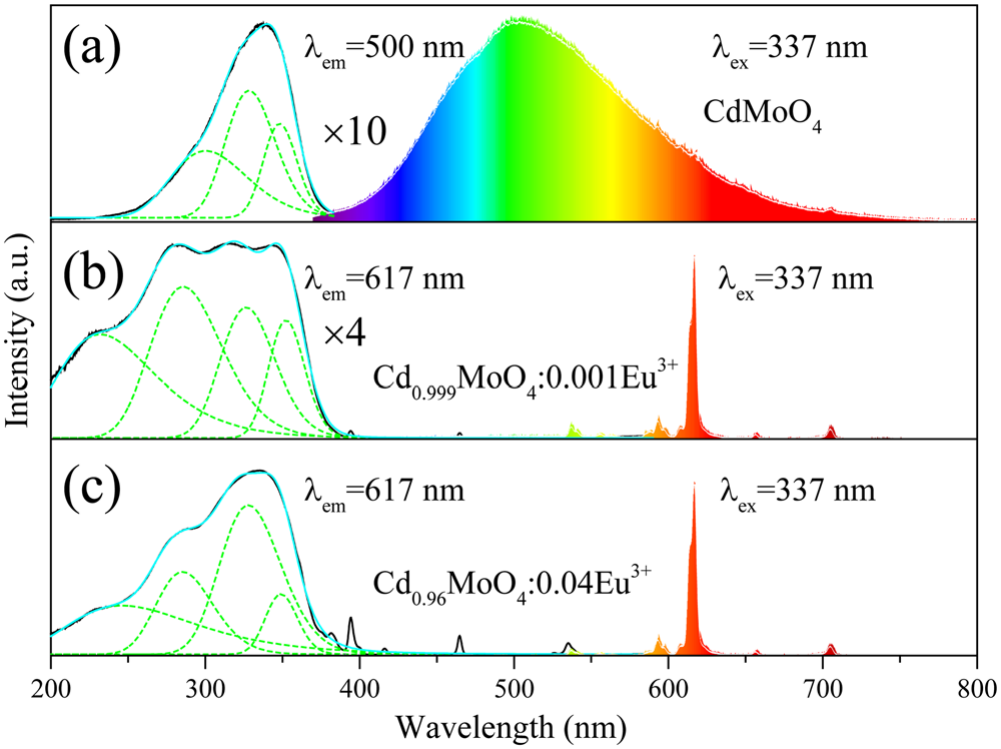
PL and PLE spectra of CdMoO_4_ (**a**); Cd_0.999_MoO_4_:0.001Eu^3+^ (**b**) and Cd_0.96_MoO_4_:0.04Eu^3+^ (**c**) phosphors.

From Fig. 7, PL and PLE spectra of CdMoO_4_, Cd_0.999_MoO_4_:0.001Eu^3+^ and Cd_0.96_MoO_4_:0.04Eu^3+^ phosphors are presented in an immediate contrast. In Fig. 7(a), pure CdMoO_4_ sample exhibits a strong green emission band with a maximum at about 504 nm under 337 nm excitation. When monitoring at 504 nm, the PLE spectrum in Fig. 7(a) indicated that the as-synthesized CdMoO_4_ phosphor exhibits a broad excitation band in the range of 250–380 nm. Three obvious excitation peaks for CdMoO_4_ host were observed at room temperature, which can be further deconvoluted by assuming a Gaussian type profile into the three excitation peaks at 300 nm, 328 nm and 348 nm, which are attributed to the charge transfer transition of O^2−^ → Mo^6+^ and O^2−^ → Cd^2+^ transitions.

As for the Eu^3+^ singly doped CdMoO_4_ sample, its PL and PLE spectra are presented in Fig. 7b and c. The PL spectrum under the excitation of 337 nm has a series of sharp line emissions at 594, 617,657 and 705 nm, due to the ^5^D_0_ − ^7^F_J_ (J = 1, 2, 3 and 4) characteristic transitions of Eu^3+^ ions. The emission peak of CdMoO_4_ host could not be observed owing to the energy transfer from CdO_8_ and MoO_4_ polyhedra to Eu^3+^ ions. In the excitation spectrum of CdMoO_4_:Eu^3+^ phosphors monitoring at ^5^D_0_ − ^7^F_2_ emission of Eu^3+^ ions, the broad absorption band from 200 to 380 nm which was decomposed into four components by Gaussian fitting can be assigned to the combination of the charge transfer (CT) transitions. Compared with the excitation spectra of CdMoO_4_ host, the new excitation band at about 246 nm was observed. From Figs 6 and 7b and c it can be seen that the excitation band at about 328 nm enhanced sharply. This is explained by the fact that the CdO_8_ and MoO_4_ polyhedra in three-dimensional network structure can transfer energy to Eu^3+^ ions.

**Figure 8:**
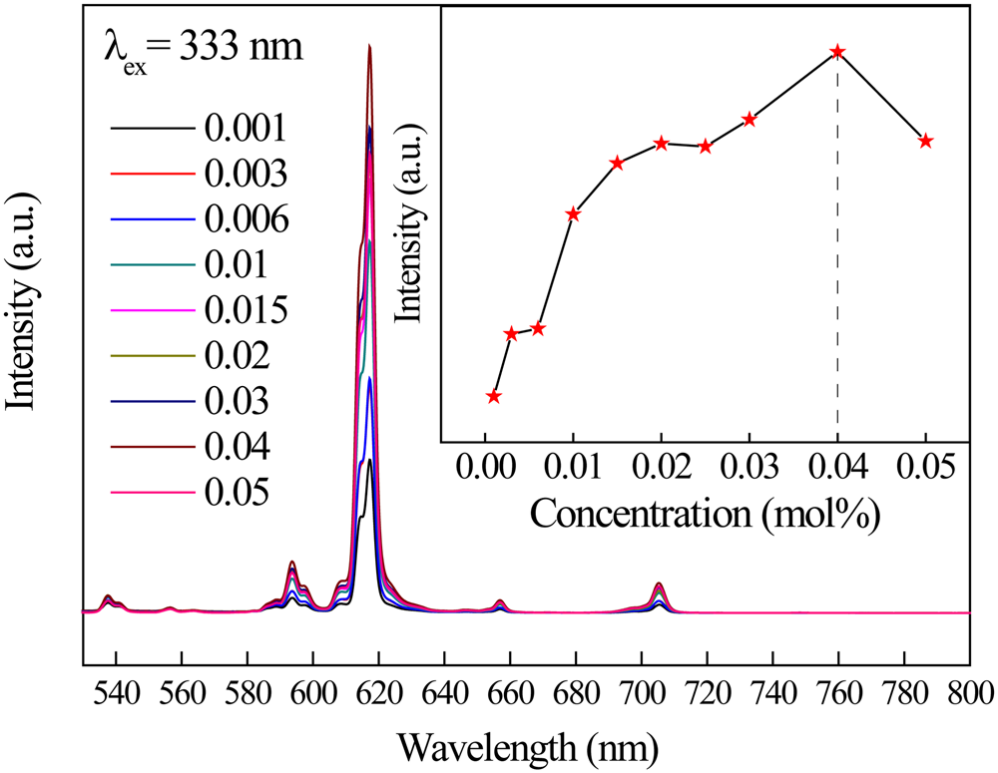
The emission spectra of Cd_1−x_MoO_4_:xEu^3+^ phosphors excited at about 337 nm in room temperature.

Figure 8 illustrates the emission spectra of Cd_1−x_MoO_4_:xEu^3+^ (x = 0.001, 0.003, 0.006, 0.01, 0.015, 0.02, 0.03, 0.04, 0.05) phosphors excited at about 337 nm. It can be seen from the picture that the strongest emission peak is at about 617 nm which is caused by the ^5^D_0_ → ^7^F_2_ electric dipole transition of Eu^3+^ ions. For the Cd_1−x_MoO_4_:xEu^3+^ phosphor, the emission intensities increased with the Eu^3+^ concentration up to 0.04, and then decreases with increasing concentration due to the concentration quenching.

**Figure 9:**
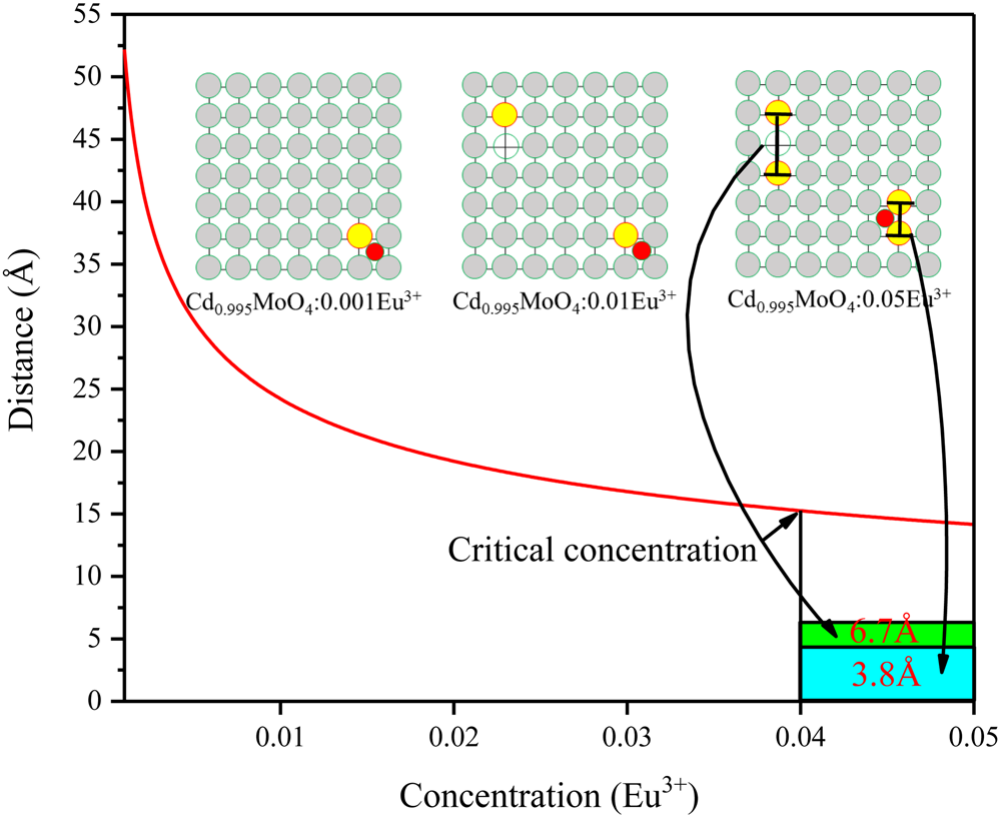
The mechanism of concentration quenching in Eu^3+^ doped CdMoO_4_:Eu^3+^ phosphor.

When Eu^3+^ ions are doped into the CdMoO_4_ host, with the increase of Eu^3+^ doping concentration, the relative distance between Eu^3+^ ions will be reduced gradually. Their distance (${\rm{d}}$) can be written as:

4 $${\rm{d}}=2\times \sqrt[3]{\frac{3\times V}{4\times \pi \times c\times N}}$$

where ${\rm{V}}$ is the volume of the unit cell, ${\rm{c}}$ is the critical concentration of the activator ion, and N is the number of host cations in the unit cell. For the CdMoO_4_ host, by taking the values of N = 4 (N = Z × 1, Z is the number of formula per unit cell) and V = 297.64 Å^3^, the relative distance between Eu^3+^ ions (${\rm{d}}$) varies with the doping concentration (${\rm{c}}$) are as shown in Fig. 9.

When the doping concentration of Eu^3+^ is low, Eu^3+^ ions are dispersed in the CdMoO_4_ host. The relative distance decreases sharply with the increase of doping concentration. When the concentration of doped Eu^3+^ is more than the critical concentration, it will no longer follow the Equation (4) due to the defect reaction in Equations (5 and 6). As a result, some of Eu^3+^ ions will enter into the adjacent lattice sites and share a point defect (${V^{\prime\prime} }_{Cd}$ or ${O^{\prime\prime} }_{i}$) to achieve charge balance. The defect equations are as follows:

5 $$E{u}_{2}{O}_{3}\,\mathop{\longrightarrow }\limits^{CdMo{O}_{4}}\,2E{u}_{Cd}^{\,.}+{O^{\prime\prime} }_{i}+2{O}_{O}$$

6 $$E{u}_{2}{O}_{3}\,\mathop{\longrightarrow }\limits^{CdMo{O}_{4}}\,2E{u}_{Cd}^{\,.}+{V^{\prime\prime} }_{Cd}+3{O}_{O}$$

Combined with the crystal structure, we believe that the position of point defects are as shown in Fig. 9. In CdMoO_4_:Eu^3+^ phosphor, Eu^3+^ is an isolated emission center which has been reported that the typical critical distance is about 5 Å. That is to say, the exchange interaction becomes effective when the Eu^3+^ − Eu^3+^ distance is shorter than 5 Å. When the concentration of Eu^3+^ is low, the interaction of Eu^3+^ − Eu^3+^ can be almost neglected because of the long distance between Eu^3+^ ions. However, the relative distance is only about 3.8 Å when two Eu^3+^ ions occupy adjacent Cd sites. This indicates that the mechanism of exchange interaction plays an important role in energy transfer between the adjacent Eu^3+^ ions in CdMoO_4_:Eu^3+^ phosphor. And the point defect of ${O^{\prime\prime} }_{i}$ is also detrimental to the luminescence of Eu^3+^ ions. This is the reason why concentration quenching occurred.

**Figure 10:**
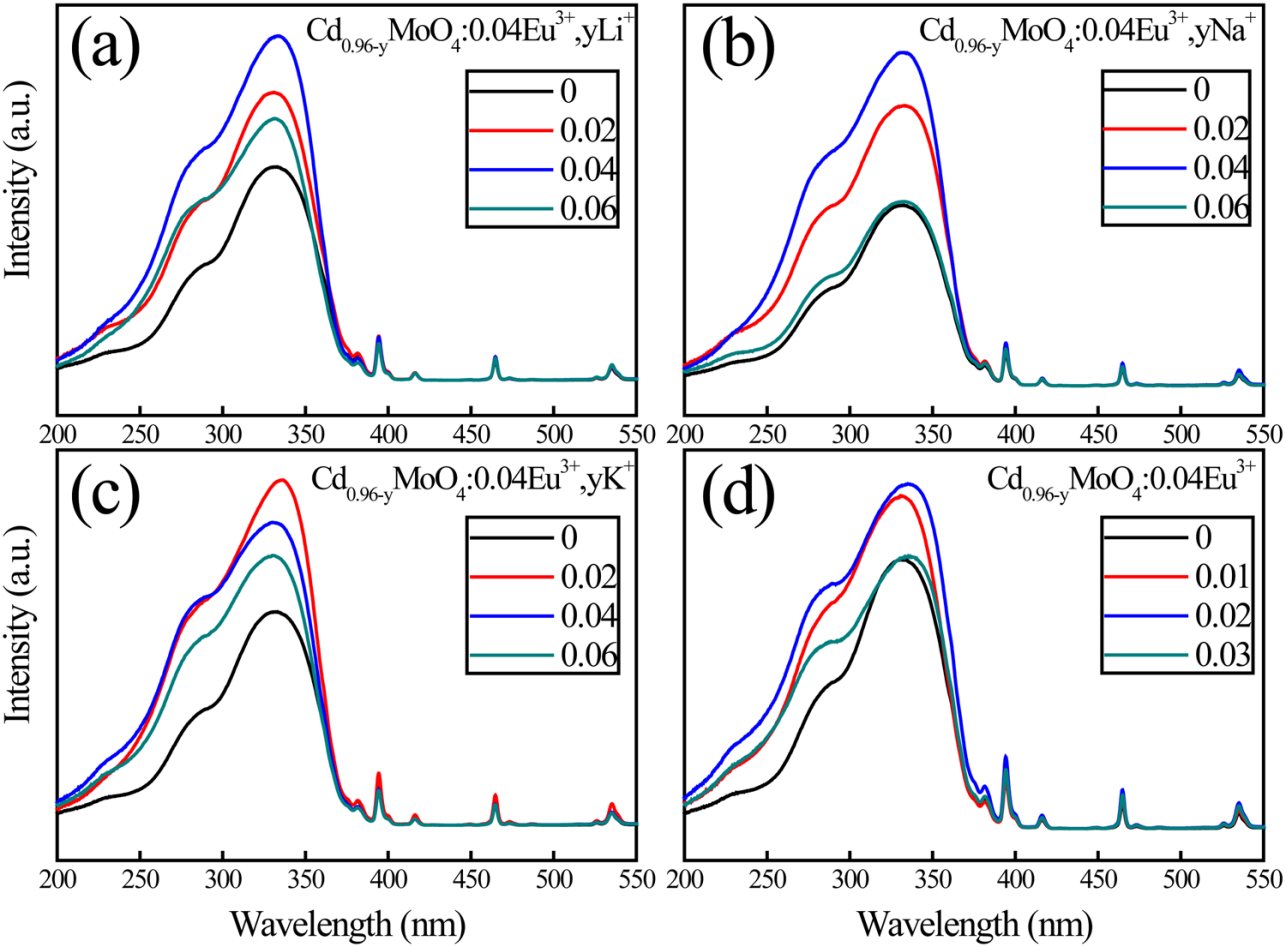
Excitation spectra of Cd_0.96−y_MoO_4_:0.04Eu^3+^, yM (M = Li^+^, Na^+^, K^+^ ions) and Cd_0.96−y_MoO_4_:0.04Eu^3+^ phosphors monitoring at 617 nm in room temperature.

The charge imbalance caused by trivalent Eu^3+^ ions occupying divalent Cd^2+^ sites in the CdMoO_4_ host affects the intensity of PL spectra. We believe that the distance between Eu^3+^ ions can be regulated by charge compensation^25^. Therefore, the variation of PL intensity with different charge compensator doping concentrations is displayed in Fig. 10. As can be observed in this picture, the effect of charge compensators, Li^+^, Na^+^, K^+^ ions and Cd^2+^ vacancy, on red emission of Eu^3+^ is enhanced markedly. The photoluminescence intensity of Cd_0.96−y_MoO_4_:0.04Eu^3+^, yLi^+^, Cd_0.96−y_MoO_4_:0.04Eu^3+^, yNa^+^ and Cd_0.96−y_MoO_4_:0.04Eu^3+^ phosphors reached the maximum when the charge is balanced. This indicates that the charge imbalance affects the photoluminescence properties of Eu^3+^ ions in CdMoO_4_ host. The maximum PL intensity is reached when the concentration of K^+^ ions in Cd_0.96−y_MoO_4_:0.04Eu^3+^, yK^+^ is 0.02. This is caused by the presence of lattice distortion that affects the photoluminescence properties. The lattice expansion corresponds to the larger ionic radius of K^+^ (1.51 Å) than that of Cd^2+^ (1.10 Å).

**Figure 11:**
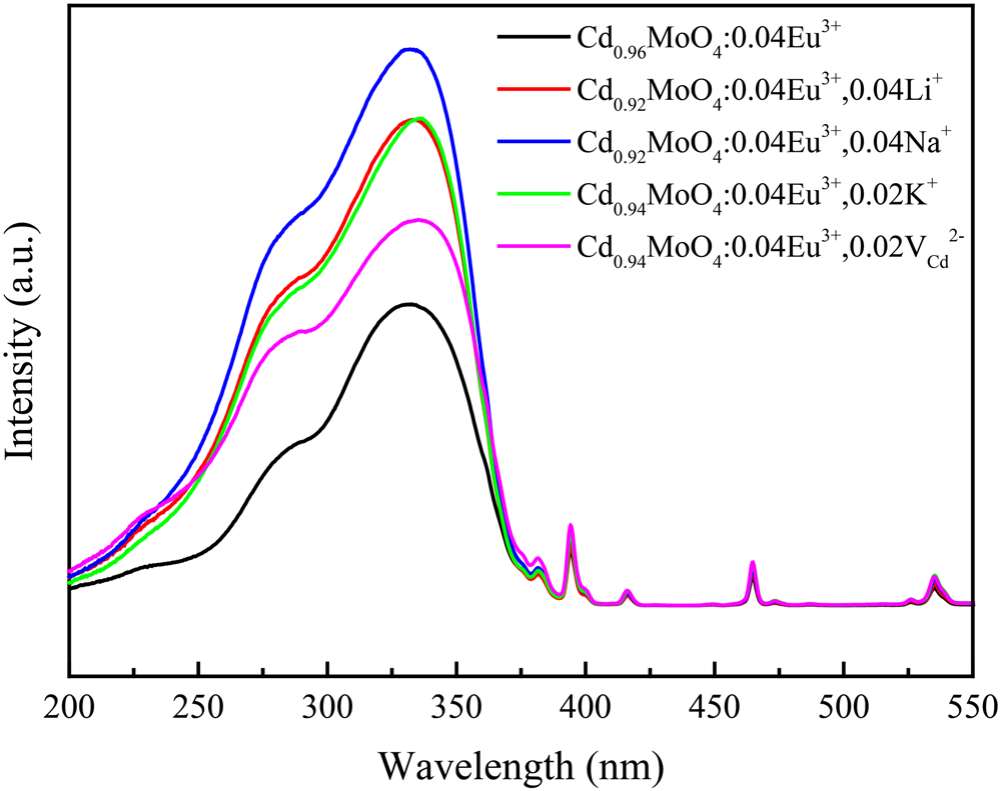
Excitation spectra of Cd_0.96−y_MoO_4_:0.04Eu^3+^, yM (M = Li^+^, Na^+^, K^+^ ions and Cd^2+^ vacancy) phosphors.

From above we can see that both Li^+^, Na^+^, K^+^ and Cd^2+^ vacancies can significantly enhance the fluorescence intensity of CdMoO_4_:Eu^3+^ phosphors. In order to compare the photoluminescence enhancement of different charge compensators, the excitation spectra of Cd_0.96−y_MoO_4_:0.04Eu^3+^, yM (M = Li^+^, Na^+^, K^+^ ions and Cd^2+^ vacancy) phosphors were shown in Fig. 11. Selected concentrations of charge compensator are the optimum doping concentration of Li^+^, Na^+^, K^+^ ions and Cd^2+^ vacancies. As can be seen from Fig. 11, the photoluminescence intensity is increased by two times when Na^+^ ions were introduced as charge compensator. So why a small amount of charge compensator can greatly improve the fluorescence intensity?

### The Mechanism of Charge Compensation

**Figure 12:**
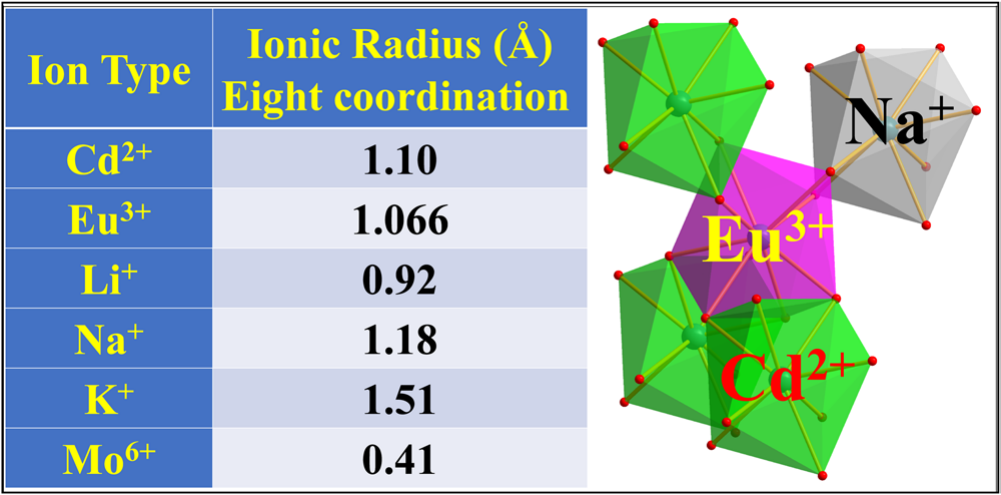
Ionic radius and relative positions of doping ions.

Figure 12 shows the radius and relative position of doping ions in charge compensated CdMoO_4_:Eu^3+^ system. It can be seen that the ionic radius of Eu^3+^ and Cd^2+^ ions are approximately equal. Therefore, Eu^3+^ can successfully occupy the Cd^2+^ lattice. On the basis of this theoretical development, Li^+^, Na^+^, K^+^ ions and Cd^2+^ vacancies were introduced as a charge compensators because a large number of lattice defects appeared with the introduction of Eu^3+^ ions.

**Figure 13:**
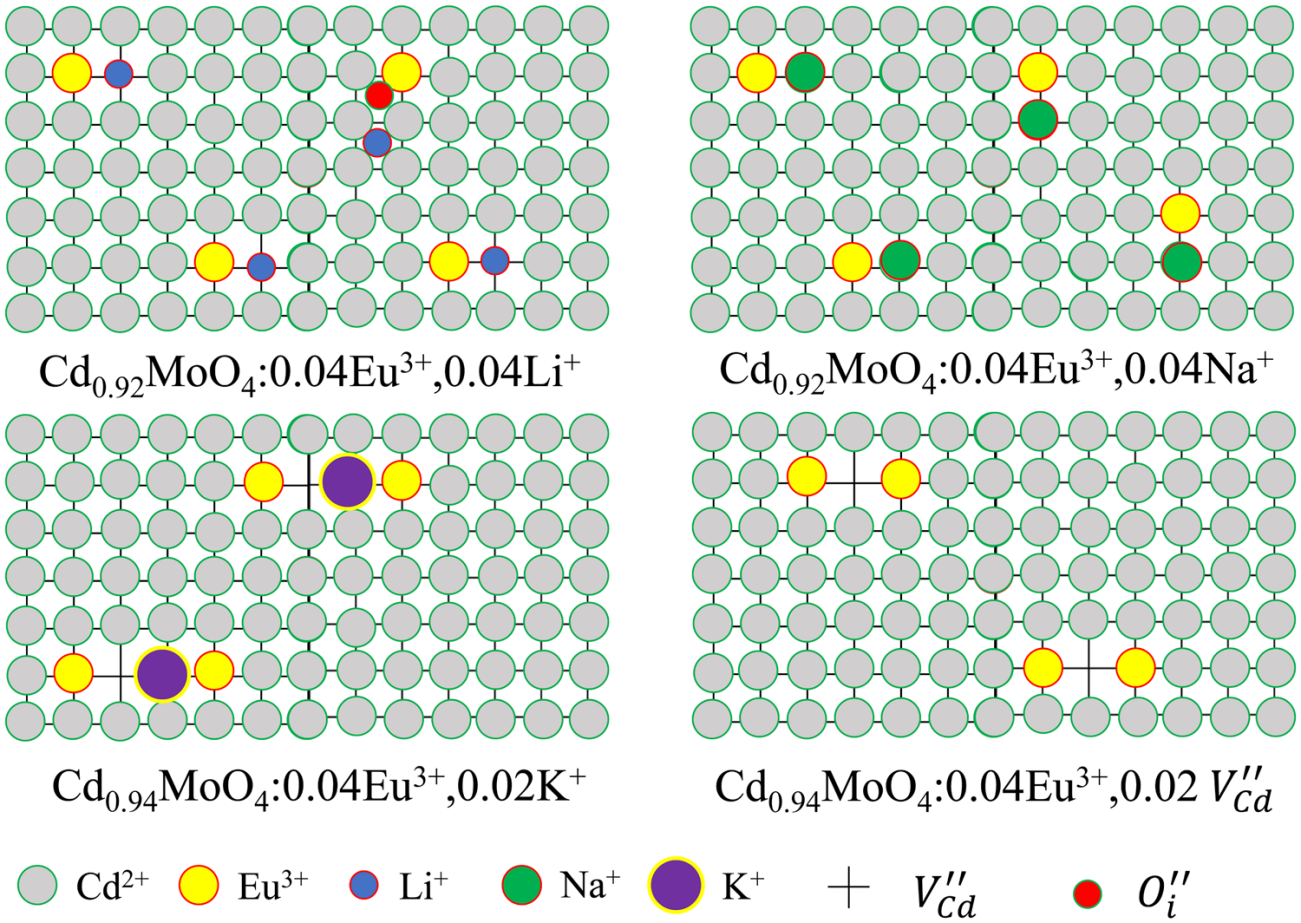
The schematic diagram of luminescence enhancement mechanisms with different charge compensators.

The possible relative position of doping ions in CdMoO_4_ host and photoluminescence enhancement mechanism is shown in Fig. 13. The diagram explains how the introduced Li^+^, Na^+^, K^+^ ions and Cd^2+^ vacancy break the interaction of Eu^3+^ ions. The point defect reaction of the cation sublattice was written as Equations (5 and 6). In particular, a certain amount of interstitial occupation of O atoms (${O^{\prime\prime} }_{i}$) is introduced into the lattice due to the charge imbalance by Eu^3+^/Cd^2+^. This leads to the increase of cell parameters and the decrease of luminescence intensity of Eu^3+^ ions. Due to the smaller ion radius of Li^+^ ions, some of interstitial occupation of doping Li^+^ ions ($L{i}_{i}^{\,.}$) and O^2−^ ions (${O^{\prime\prime} }_{i}$) appear in the Cd_0.92_MoO_4_:0.04Eu^3+^, 0.04Li^+^ sample. The point defect reaction of the cation sublattice was written as Equation (7). Therefore, the interstitial occupation of Li^+^ ions increased the cell parameters. When the concentration of Li^+^ ions increased more than 0.04, A large amount of ($L{i}_{i}^{\,.}$) and (${O^{\prime\prime} }_{i}$) will appear to reduce the photoluminescence intensity of Eu^3+^ ions. The point defect reaction of the K^+^ cation sublattice may be written as Equation (8). The large ionic radius of K^+^ ions resulted in the highest lattice distortion in the sample. As can be seen in Fig. 13, the lattice shrinkage induced by the point defect of $E{u}_{Cd}^{\,.}$ and ${V^{\prime\prime} }_{Cd}$ were compensated by the great lattice expansion of the ${K^{\prime} }_{Cd}$ defect. The appropriate concentration of K^+^ ions gives the best optical performance. Because the appearance of $E{u}_{Cd}^{\,.}$ − ${V^{\prime\prime} }_{Cd}$ pair can stabilize the luminescent environment of the $E{u}_{Cd}^{\,.}$ − ${K^{\prime} }_{Cd}$ pair. The point defects equation with Cd^2+^ vacancy is Equations (9 and 10). Therefore, the introduction of ${V^{\prime\prime} }_{Cd}$ reduced the point defect of ${O^{\prime\prime} }_{i}$ and thus increased the photoluminescence intensity.

The ionic radius of Na^+^ ions is most approaching to Cd^2+^, and the introduction of Na^+^ ions can produce a negative charge. The introduction of Na^+^ ions can also reduce the structural distortion well because of the suitable ionic radius. This results in the concentration decrease of ${O^{\prime\prime} }_{i}$ and thus decrease the cell parameters. Therefore, as a charge compensator, Na^+^ ions have an ideal ion radius and charge states. The point defect reaction of the Na^+^ cation sublattice may be written as Equation (11). Eu^3+^ and Na^+^ ions will occupy the adjacent Cd^2+^ ion lattice sites in order to achieve charge balance and reduce the lattice distortion. Therefore, the introduction of Na^+^ ions can compensate the effect of the charge imbalance well, which can significantly enhance the luminescence intensity of Eu^3+^ ions.

7 $$E{u}_{2}{O}_{3}+L{i}_{2}C{O}_{3}\,\mathop{\longrightarrow }\limits^{CdMo{O}_{4}}\,2E{u}_{Cd}^{\,.}+L{i^{\prime} }_{Cd}+L{i}_{i}^{\,.}+{O^{\prime\prime} }_{i}+4{O}_{O}+C{O}_{2}\,\uparrow $$

8 $$2E{u}_{2}{O}_{3}+{K}_{2}C{O}_{3}\,\mathop{\longrightarrow }\limits^{CdMo{O}_{4}}\,4E{u}_{Cd}^{\,.}+2{K^{\prime} }_{Cd}+{V^{\prime\prime} }_{Cd}+7{O}_{O}+C{O}_{2}\,\uparrow $$

9 $$Mo{O}_{3}\,\mathop{\longrightarrow }\limits^{CdMo{O}_{4}}\,{V^{\prime\prime} }_{Cd}+{V}_{O}^{\mathrm{.}.}+3{O}_{O}$$

10 $$E{u}_{2}{O}_{3}+Mo{O}_{3}\,\mathop{\longrightarrow }\limits^{CdMo{O}_{4}}\,2E{u}_{Cd}^{\,.}+{V^{\prime\prime} }_{Cd}+M{o}_{Mo}+6{O}_{O}$$

11 $$E{u}_{2}{O}_{3}+N{a}_{2}C{O}_{3}\,\mathop{\longrightarrow }\limits^{CdMo{O}_{4}}\,2E{u}_{Cd}^{\,.}+2N{a^{\prime} }_{Cd}+4{O}_{O}+C{O}_{2}\,\uparrow $$

**Figure 14:**
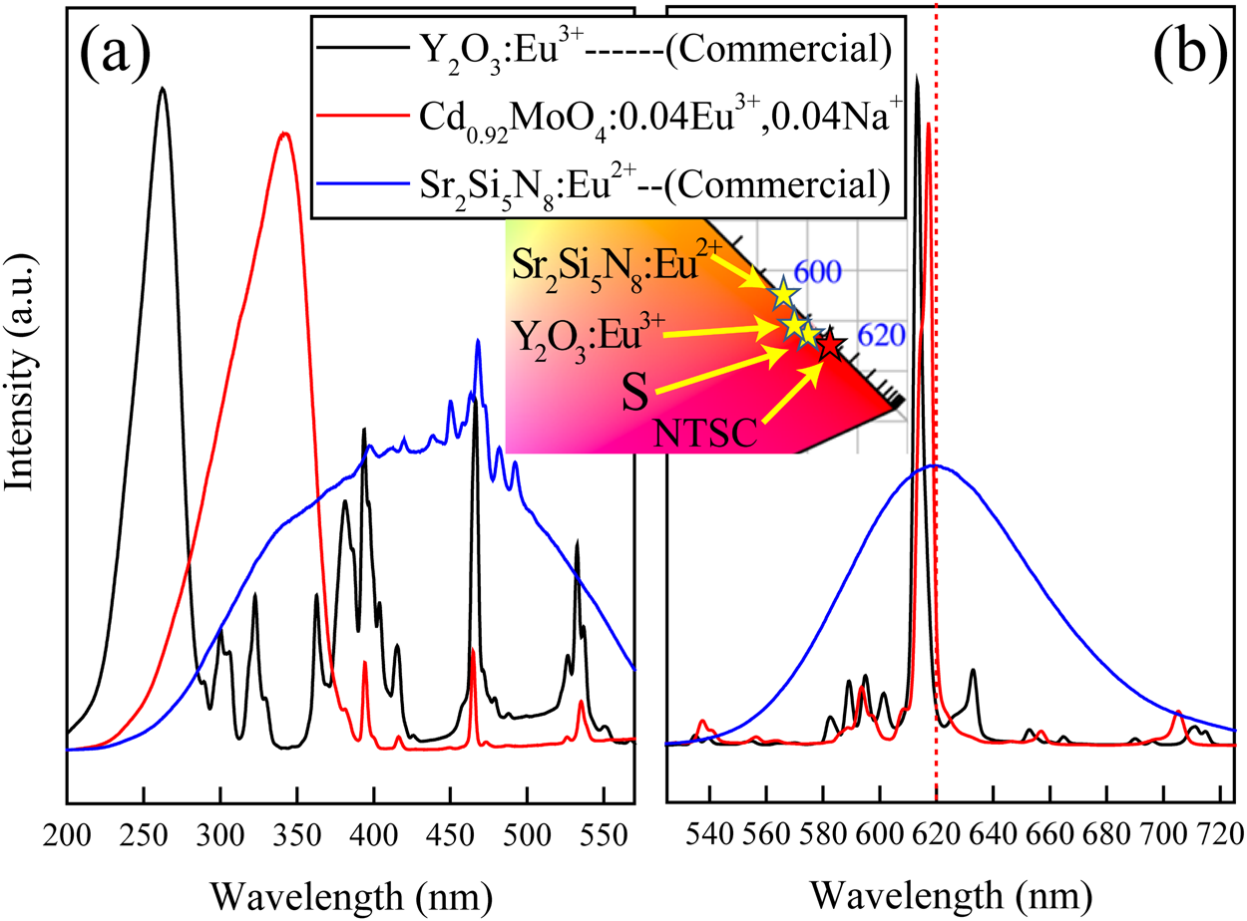
The PLE (**a**), PL (**b**) spectra and chromaticity coordinates in Commission Internationale de l′E′clairage (CIE) 1931 color spaces (insert) of prepared Cd_0.92_MoO_4_:0.04Eu^3+^, 0.04Na^+^ phosphor compared with those of commercial red-emitting phosphor (Y_2_O_3_:Eu^3+^ and Sr_2_Si_5_N_8_:Eu^2+^).

With the purpose of application for white-light LEDs with near-UV based chips as excitation sources, photoluminescence properties of Cd_0.92_MoO_4_:0.04Eu^3+^, 0.04Na^+^ and commercial red emission phosphors are compared. Figure 14a and b shows the intuitive compared PL and PLE spectra of Cd_0.92_MoO_4_:0.04Eu^3+^, 0.04Na^+^ and commercial Y_2_O_3_:Eu^3+^ and Sr_2_Si_5_N_8_:Eu^2+^ red emitting phosphors under the same measurement conditions. According to the compared spectra, the intensity of the broad excitation band at about 345 nm and sharp emission peak at about 617 nm in Cd_0.92_MoO_4_:0.04Eu^3+^, 0.04Na^+^ phosphors were high enough to be comparable to commercial red-emitting phosphors. The ^5^D_0_ − ^7^F_2_ emission intensity of Cd_0.92_MoO_4_:0.04Eu^3+^, 0.04Na^+^ is about 94% of Y_2_O_3_:Eu^3+^ commercial phosphor. The CIE chromaticity diagram of prepared Cd_0.92_MoO_4_:0.04Eu^3+^, 0.04Na^+^ and commercial red emitting phosphors was shown in the insert of Fig. 14. It can be seen that the optimal Cd_0.92_MoO_4_:0.04Eu^3+^, 0.04Na^+^ approaches the National Television System Committee (NTSC) ideal red color (x = 0.67, y = 0.33) and it is much closer to the red region on the CIE chromaticity diagram than commercial Sr_2_Si_5_N_8_:Eu^2+^ and Y_2_O_3_:Eu^3+^ phosphors. Thus, as-prepared Cd_0.92_MoO_4_:0.04Eu^3+^, 0.04Na^+^ phosphor is promising red-emitting phosphor for near-UV- LEDs.

## Conclusion

In summary, a series of Eu^3+^ and alkali metal ions co-doped CdMoO_4_ phosphors were prepared by the solid-state reaction method in air atmosphere. In CdMoO_4_ crystal structure, CdO_8_ and MoO_4_ polyhedra were connected with each other by coplanar to form a 3D network. Eu^3+^ ions occupied Cd^2+^ sites will be excited with the energy collected by 3D network structure. In addition, eight adjacent MoO_4_ polyhedra can also effectively transfer energy to the Eu^3+^ ion. However, the interaction between Eu^3+^ ions is further amplified in the 3D network structure. The introduced Li^+^, Na^+^, K^+^ ions and Cd^2+^ vacancy which can avoid ${O^{\prime\prime} }_{i}$ will separate the couple of Eu^3+^ ions and break the interaction between them. Since the suitable ionic radius and valence states, the introduction of Na^+^ can break the energetic interaction of Eu^3+^ ions through the 3D network structure. This is because the introduction of Na^+^ ions can decrease the lattice distortion and avoid the appearance of ${O^{\prime\prime} }_{i}$ point defect. This mechanism provides a model how to use charge compensator to break the energetic interaction between Eu^3+^ ions which expected to be used to design efficient luminescent materials.

## Experiment

### Materials and Synthesis

A series of Eu^3+^ doped CdMoO_4_ phosphors were synthesized by a traditional high temperature solid state reaction in air atmosphere. Stoichiometric amounts of CdO (99.9%), Eu_2_O_3_ (99.99%) and MoO_3_ (99%) were mixed homogeneously in an agate mortar. The mixtures were put into an alumina crucible and calcined in the muffle furnace at 800 °C for 3 hours in air atmosphere, and then the white powder phosphor was obtained. In some cases, appropriate amounts of Li_2_CO_3_ (99.9%), Na_2_CO_3_ (99.9%) and K_2_CO_3_ (99.9%) were added as charge compensators. In order to reduce the amount of Cd in the sample and increase the concentration of Cd vacancy, the stoichiometry of CdO is intentionally reduced. All samples were ground into a powder in an agate mortar and pestle for further analysis.

### Experimental methods

The phase purity was verified by the powder X-ray diffraction (XRD) measurement performed on a Bruker D8 Advance X-ray diffractometer (Cu Kα_1_ radiation, λ = 0.15406 nm) and high-resolution X-ray diffraction was recorded over an angular (2θ) range of 5–130° with radiation at a 0.02^o^ (2θ)/s scanning step. Structural refinements of X-ray diffractograms were performed by the GSAS2 program. UV-Vis diffuse reflectance spectra (DRS) were collected using a V-670 (JASCO) UV-Vis spectrophotometer. The photoluminescence excitation (PLE) and emission (PL) spectra were recorded with a Hitachi F-4600 spectrophotometer equipped with a 150 W xenon lamp as an excitation source at room temperature. DFT calculations on the CdMoO_4_ host was carried out by using the CASTEP code^21^. All the measurements were performed at room temperature.

### Details of calculation

The CASTEP program was employed to determine the Band structure and Orbital population by density functional theory^26–28^. The Vanderbilt ultrasoft pseudopotential with a cutoff energy of 380 eV was used to ensure the precision of the results. Brillouin zone integration was represented using the K-point sampling scheme of 3 × 3 × 1 Monkhorst−Pack scheme^29^. At first, the Broyden, Fletcher, Goldfarb, Shannon (BFGS) method was used to achieve the geometry optimization. Atomic positions and lattice parameters were optimized simultaneously during the geometry optimization. The convergence tolerance for geometry optimization was selected with the differences in total energy (5.0 × 10^−6^ eV/atom), the maximal ionic Hellmann–Feynman force (1.0 × 10^−2^ eV/Å), the stress tensor (2.0 × 10^−2^ GPa), and the maximal displacement (5.0 × 10^−4^ Å). The calculations were conducted within the generalized gradient approximations (GGA), using the exchange and correlation functional. The convergence criterion for the self-consistent field (SCF) was set to 5.0 × 10^−7^ eV/atom in whole processes.
